# Donor cord blood aging accelerates in recipients after transplantation

**DOI:** 10.1038/s41598-023-29912-2

**Published:** 2023-02-14

**Authors:** Makoto Onizuka, Tadashi Imanishi, Kaito Harada, Yasuyuki Aoyama, Jun Amaki, Masako Toyosaki, Shinichiro Machida, Eri Kikkawa, Sanetoshi Yamada, Kazuhiko Nakabayashi, Kenichiro Hata, Ken Higashimoto, Hidenobu Soejima, Kiyoshi Ando

**Affiliations:** 1 Department of Hematology and Oncology, Tokai University School of Medicine, Isehara, Kanagawa, 259-1143 Japan; 2 Department of Molecular Life Science, Tokai University School of Medicine, Isehara, Kanagawa, 259-1143 Japan; 3 Department of Maternal-Fetal Biology, Research Institute, National Center for Child Health and Development, Tokyo, 157-8535 Japan; 4 Department of Molecular and Cellular Biology, Gunma University Graduate School of Medicine, Gunma, 371-8511 Japan; 5 Division of Molecular Genetics and Epigenetics, Department of Biomolecular Sciences, Faculty of Medicine, Saga University, Saga, 849-8501 Japan

**Keywords:** Stem cells, Ageing, Medical research, Stem-cell research

## Abstract

Cord blood stem cell transplantation is an important alternative for patients needing hematopoietic stem cell transplantation. However, it is unclear how cord blood cells, which are 0 years old, age in the recipient’s body after allogeneic transplantation. We performed DNA methylation (DNAm) age analysis to measure the age of cells using post-transplant peripheral blood in 50 cases of cord blood transplantation. The median chronological age (the time elapsed from the date of the cord blood transplant to the day the sample was taken for DNAm analysis) of donor cells was 4.0 years (0.2–15.0 years), while the median DNAm age was 10.0 years (1.3–30.3 years), and the ratio of DNAm age to chronological age (AgeAccel) was 2.7 (1.2–8.2). When comparing the mean values of AgeAccel in cord blood transplant cases and controls, the values were significantly higher in cord blood transplant cases. The characteristics of patients and transplant procedures were not associated with AgeAccel in this analysis, nor were they associated with the development of graft-versus-host disease. However, this analysis revealed that transplanting 0-year-old cord blood into a recipient resulted in cells aging more than twice as quickly as the elapsed time. The results shed light on the importance of the mismatch between cord blood stem cells and donor environmental factors in stem cell aging.

## Introduction

Allogeneic hematopoietic stem cell transplantation is a very powerful treatment that can cure blood diseases. These transplants offer a window into how donor cells age in the recipient’s body after transplantation and whether stem cell aging is affected by changes in their environment. It provides an important step in understanding how stem cells age^1–4^. These findings in stem cell biology are indispensable to develop better regenerative medicine procedures.

Changes in telomere length and epigenetic DNA alterations have allowed for analysis of the aging mechanism of donor cells after transplantation^1,2^; however, in the past 10 years, DNA methylation sites closely related to temporal age have been revealed, making it possible to estimate age from DNA^3,5^. This new method has revealed that cellular aging is accelerated by various diseases, compared to that in healthy people^6,7^.

DNA methylation analysis of donor cell age in patients with hematopoietic stem cell transplantation has been reported^8–10^. The DNA methylation (DNAm) age of the donor from bone marrow (BM) or peripheral blood stem cells (PBSCs) after transplantation is not affected by the recipient’s age and is reported to be approximately equivalent to the donor’s age.

In a DNAm age analysis by Horvath clock, PBSC transplantation primed by granulocyte colony-stimulating factor (G-CSF) significantly reduced the DNAm age of the donor cells compared to the chronological age of the donor but tended to increase cell age in BM transplant donors^11^. Thus, it has been shown that the DNAm age of donor cells after transplantation may differ depending on the collection method.

Cord blood is a widely used alternative transplant source. To the best of our knowledge, DNAm age analysis in cord blood transplant cases has not been performed to date. In contrast to that of donor sources such as BM or PBSCs, the DNA methylation age of cord blood nucleated cells has been shown to be 0 years^5^. Therefore, unlike other donor sources, the donor age at the time of transplantation is unified to 0 years. For this reason, no donor-dependent factors other than sex, number of injected cells, and human leukocyte antigen (HLA) matching that affect transplant treatment outcomes have been reported.

Does cord blood, which is 0 years old at the time of transplantation, change in age over time in the patient’s bone marrow? If so, what factors affect this, and can this be evaluated by DNA methylation? The purpose of our study is to clarify the extent to which the DNAm age differs from the chronological cord blood age in the patient’s body and to clarify whether there is a relationship between the occurrence of graft-versus-host disease (GVHD), a post-transplant complication, the recipient background, and DNAm age.

## Materials and methods

### Sampling and methylation analysis

Methylation analysis of DNA obtained from peripheral blood nucleated cells was performed in 54 cases after cord blood transplantation. Among them, chimerism analysis between donors and recipients was performed by the short tandem repeat (STR) method; 50 recipients and 95% of donors were subjected to analysis^12^. Transplant information for the 50 cases is shown in Table 1. Peripheral blood was collected from the cases between February 25, 2021, and December 27, 2021.

**Table 1:** Patient and control characteristics.

Characteristics	
*Age variables (years), median (range)*	
Recipient age at transplantation	59.4 (16.7–71.3)
Donor chronological age	4.0 (0.2–15.0)
Donor DNAm age	10.0 (1.0–30.3)
Donor AgeAccel*	2.7 (1.2–8.2)
Control age	10.3 (1.3–16)
Control DNAm age	8.0 (1.5–22.6)
Control AgeAccel*	0.93 (0.7–1.5)
*Sex (n)*	
Recipient	
Male	34
Female	16
Donor	
Male	31
Female	19
Control	
Male	7
Female	8
*Donor/recipient sex match (n)*	
Male to Male	17
Female to Female	2
Female to Male	17
Male to Female	14
*Conditioning (n)*	
RIC	32
MAC	18
*Disease (n)*	
ALL	7
AML	23
MDS	15
ATLL	1
CML	1
HD	1
NHL	1
SAA	1
TNC, median (range), × 10^8^/kg	2.1 (1.1–4.6)
CD34^+^ cell, median (range), × 10^5^/kg	1.1 (0.5–2.5)
*HLA disparity*	
HVG direction	
0	1
1	11
2	27
3	11
GVH direction	
0	1
1	11
2	30
3	11

*RIC* reduced intensity conditioning; *MAC* myeloablative conditioning; *ALL* acute lymphoblastic leukemia; *AML* acute myeloid leukemia; *MDS* myelodysplastic syndrome; *ATLL* adult *T*-cell lymphoma/leukemia; *CML* chronic myeloid leukemia; *HD* Hodgkin lymphoma; *NHL* non-Hodgkin lymphoma; *SAA* severe aplastic anemia; *TNC* total nuclear cell; *HVG* host-versus-graft; *GVH* graft-versus-host.

*AgeAccel = (DNAm age)/(Chronological age).

The 15 controls were examined by the National Center for Child Health and Development and Saga University for common cold or other minor symptoms. The control specimens were prepared by extracting DNA from peripheral blood nucleated cells.

The age of each control individual was checked and recorded at the time of blood sampling. Genomic DNA was extracted from 1 ml of peripheral blood using QIAamp DNA Blood Midi Kit (Qiagen). Genome-wide DNA methylation profiles of genomic DNA from the peripheral blood of control individuals (n = 15) were obtained using an Illumina Infinium HumanMetylation 450 BeadChip (Illumina, CA, USA) as described previously^13^. Conditioning regimens that included total body irradiation (TBI) > 8 Gy, melphalan (Mel) > 140 mg/m^2^, or busulfan (Bu) ≥ 7.2 mg/kg, were classified as myeloablative conditioning (MAC). Other regimens were classified as reduced intensity conditioning (RIC). Tacrolimus and short-term methotrexate were used for prophylaxis of GVHD. For the prevention of GVHD, methotrexate was administered at 10 mg/m^2^, 5 mg/m^2^, and 5 mg/m^2^ on days 1, 3, and 5, respectively. Further, from day 1 until the white blood cell count exceeded 1000/μl, filgrastim at 300 μg/m^2^/day or lenograstim at 5 μg/kg/day was administered.

### DNAm age calculation

DNAm age of blood from transplantation recipients was estimated using Zymo Research’s proprietary Human DNAge® Service (Zymo Research, Irvine, CA). DNAm ages of 15 control individuals, aged 1.3 to 16 years, were calculated using the original R scripts for DNAm age calculation by Horvath et al.^5^, using 336 CpG sites that were commonly used by the original Horvath clock and by Zymo Research’s DNAge® predictor.

### Statistical analysis

The elapsed time from the date of cord blood transplantation to the collection of peripheral blood was taken as donor chronological age. The ratio of DNAm age to chronological age was defined as AgeAccel. The relationship between DNAm age and the other continuous variables was determined using linear regression. The Student’s t-test was used to compare the mean values of DNAm age between the two groups. All testing was 2-sided. A *P* value of < 0.05 was considered statistically significant.

### Ethics approval

Sampling and analysis of transplant recipients were approved by the Institutional Review Board for Clinical Research of Tokai University (approval numbers: 20I-40). Sampling and analysis of control individuals were carried out with approval by the Institutional Review Board Committees of the National Center for Child Health and Development (NCCHD) of Japan (approval number: 234) and the Faculty of Medicine, Saga University (approval numbers: R3-3 and R3-8). All Written informed consent was obtained from all participants or their parents. The study was performed in accordance with approved guidelines and the principles of the Declaration of Helsinki.

## Results

### Patient, donor, and control characteristics

The median age at the time of cord blood transplantation in the 50 cases (34 men and 16 women) was 59.4 years (16.7–71.3 years), and the median age at the time of sample collection was 63.0 years (54.9–67.4 years). There were 23 cases of acute myeloid leukemia, 15 cases of myelodysplastic syndrome, 7 cases of acute lymphocytic leukemia, and one case each of adult *T*-cell leukemia, chronic myeloid leukemia, Hodgkin disease, non-Hodgkin disease, and severe aplastic anemia. This was the first transplant in 46 cases and the second transplant in 4 cases. Three of the second transplants were rescue transplants for engraftment insufficiency, and one was a second cord blood transplant for post-transplant recurrence. In the chimerism analysis using the STR method, 35 cases were 100% chimeras, with an average value of 99.7% (range; 95.9–100%). Pre-transplantation treatment, the total number of transplanted nucleated cells, CD34 positive cell count, and HLA discrepancies are shown in Table 1. Donor and control genders and ages are listed in Table 1. There were 10 cases of severe GVHD (grade II or higher) and 16 cases of chronic GVHD.

### Comparison of DNAm age and chronological age

**Figure 1:**
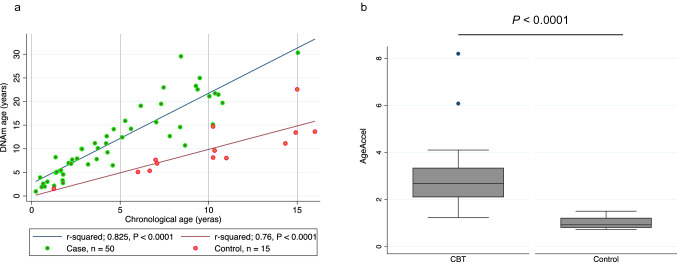
Comparison of DNAm age and chronological age in cases and controls. (**a**) While the control matched the chronological age on the horizontal axis and the DNAm age on the vertical axis, the DNAm age was higher than the chronological age in the donor after cord blood transplantation. There was a strong correlation between chronological age and DNAm age in both control and cord blood transplant cases. (**b**) The mean of AgeAccel in controls (mean; 1.01, 95% CI, 0.88–1.14) was significantly lower than cord blood transplant cases (mean; 2.84, 95% CI, 2.51–3.17) (*t*-test, *P* < 0.001).

**Figure 2:**
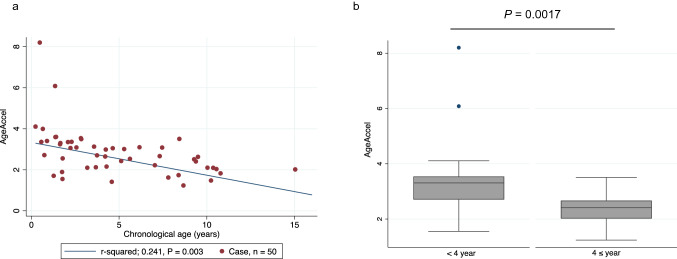
AgeAccel by time post-transplant. (**a**) The relationship between AgeAccel and chronological age was negatively correlated in cord blood transplant cases, but AgeAccel was still doubled in cases more than 10 years post-transplantation. (**b**) Cases were stratified into two groups by sample time, using a cut-off of 4 years post-transplant (the median elapsed time after transplantation), and the mean values for AgeAccel were compared using Student’s t-test. The mean AgeAccel for the group less than 4 years post-transplantation was 3.33 (95% CI, 2.77–3.90), significantly higher than the mean AgeAccel for the 4 years or more after transplantation group, which was 2.34 (95% CI, 2.09–2.59) (*P* = 0.0017).

The median donor chronological age was 4.0 years (0.2–15.0 years), while the median DNAm age was 10.0 years (1.3–30.3 years), and the AgeAccel was 2.7 (1.2–8.2). The relationship between DNAm age and chronological age was strongly correlated in cord blood transplant cases and controls (*r*-squared = 0.825, *P* < 0.0001 and *r*-squared = 0.760, *P* < 0.0001, respectively) (Fig. 1a). When comparing the mean values of AgeAccel in cord blood transplant cases and controls, the values were significantly higher in cord blood transplant cases (*P* < 0.0001) (Fig. 1b). There was no statistically significant difference between the mean AgeAccel in patients with and without acute GVHD (*P* = 0.98). Likewise, chronic GVHD revealed no statistically significant difference (*P* = 0.84). Comparison of the AgeAccel mean between donor-recipient sex differences and other combinations of female to male showed no statistically significant difference (*P* = 0.55). There was no significant association between the total number of transplanted nucleated cells and CD34-positive cells and AgeAccel (Supplementary Figsure. 1a,b). Furthermore, considering HLA concordance degrees, neither the graft-versus-host direction nor the host-versus-graft (HVG) direction showed any association between the number of mismatched alleles and AgeAccel (*P* = 0.45 and *P* = 0.24, respectively). In addition, no significant differences in AgeAccel were observed in disease or patient age at the time of transplantation. Figure 2a shows that AgeAcel is higher in the early post-transplant period. When comparing the AgeAccel mean values for those transplants with a chronological age of less than 4 years (the median chronological age), AgeAccel was significantly higher than for those of 4 years or more (*P* = 0.0017) (Fig. 2b).

## Discussion

We found that transplanted cord blood cells aged twice as fast as their chronological age in the recipient’s body. We can be certain that this was not due to recipient cell contamination, as STR analysis confirmed that peripheral blood was almost 100% donor chimerism. In our analysis of cord blood transplant cases, we believe that a major factor in cord blood cell aging is that the cells have spent several years in recipient bone marrow environments that are 16.7–71.3 years older than the naïve cord blood, which was 0 years of age.

In previous reports of DNAm analysis after allogeneic hematopoietic stem cell transplantation, the age of the donor cells was similar to the chronological age with no statistically significant deviations. This may be because previous reports have dealt with cases of related or unrelated transplantation with relatively small age differences between the donor and recipient.

Previous DNAm age analyses have studied cases of allogeneic transplants between siblings or unrelated donors, but cord blood transplants are significantly different. First, the total number of nucleated and CD34-positive cells that are hematopoietic stem cells is about 1/10th that of bone marrow transplantation and 1/100th of peripheral blood stem cell transplantation. The small number of stem cells transplanted may be a pressure for aging. This concept is supported by the finding that in peripheral blood stem cell transplantation primed with G-CSF, which has a larger number of transplanted cells, there is a tendency for the DNAm age of the recipient cells to be lower^11^. In addition, cord blood transplantation differs from other allogeneic transplantations in that the transplant is stable even when there is no complete HLA match. Allogeneic immune responses from cells remaining in the recipient after transplantation with HLA-mismatched cord blood are also considered to be age stressed. However, in this study, we compared the number of HLA-mismatched alleles in the HVG direction with AgeAccel but did not find a significant difference.

A limitation of this study was that DNAm age was measured at only one point in time, and it is necessary to confirm multiple points of DNAm age in the same case. In particular, the high value of AgeAccel in the early post-transplant period may have been due to cells dividing more actively and the excess load placed on various hematopoietic stem cells in conditions such as GVHD and infectious disease. In our study, the number of injected nucleated cells and the number of CD34-positive cells did not affect AgeAccel, but it was unclear whether this reflected the early impact of the number of transplanted cells because the sample collection point varied from case to case. Our results also show that acute and chronic GVHD were not associated with AgeAccel, but they had not necessarily developed GVHD at the point of specimen collection. In order to clarify these points, it is necessary to set the time points for sample collection in advance and evaluate all patients at the same points. It is also necessary to assess whether AgeAccel will decline over time. However, in this study, the longest period was 15 years post-transplantation, but the age analysis of peripheral blood nucleated cells in DNAm age, in this case, was 30 years old. This supports the hypothesis that the acceleration of the aging of cord blood is not a short-term phenomenon only occurring immediately after transplantation but rather that the donor cells age faster than their chronological age over a long period. The results of this study suggest that the recipient environment that nurtures stem cells affects cellular aging. In order to clarify these points, it is necessary to analyze more cases.

## Supplementary Information

Supplementary Information 1.

Supplementary Information 2.

## Data Availability

The data analyzed during this study are included in this article and its supplementary information files.
