# Supplementary material for: Donor cord blood aging accelerates in recipients after transplantation
Source: Sci Rep. 2023 Feb 14;13:2603. doi: 10.1038/s41598-023-29912-2 (PMC9929229; doi:10.1038/s41598-023-29912-2)
Supplement: Supplementary file 2 — Supplementary Information 2. [file 41598_2023_29912_MOESM2_ESM.pptx]

## Slide 1
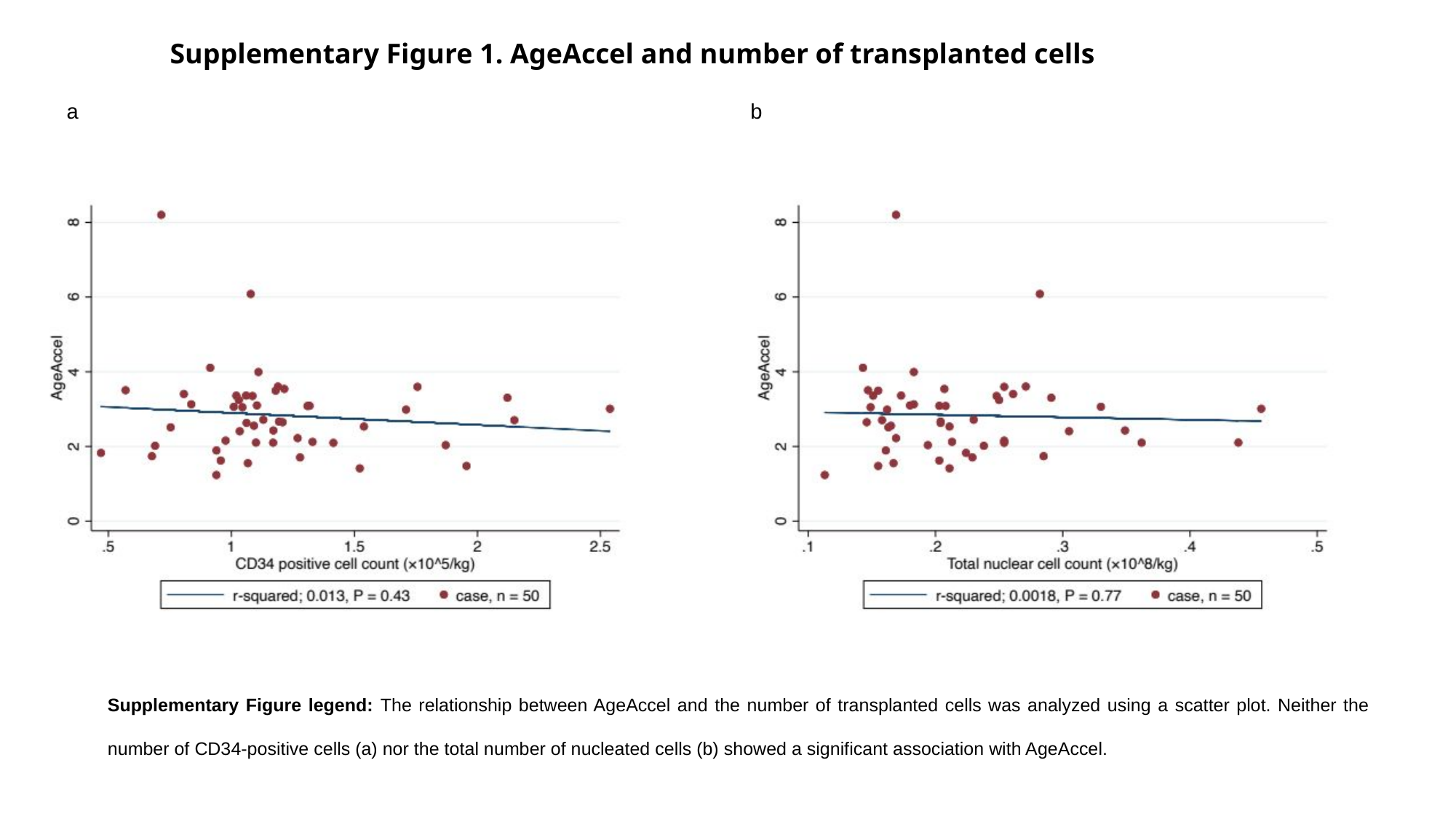

Supplementary Figure 1. AgeAccel and number of transplanted cells
b
a
Supplementary Figure legend: The relationship between AgeAccel and the number of transplanted cells was analyzed using a scatter plot. Neither the number of CD34-positive cells (a) nor the total number of nucleated cells (b) showed a significant association with AgeAccel.
